# Large-Scale Analysis of Acute Ethanol Exposure in Zebrafish Development: A Critical Time Window and Resilience

**DOI:** 10.1371/journal.pone.0020037

**Published:** 2011-05-19

**Authors:** Shaukat Ali, Danielle L. Champagne, Alia Alia, Michael K. Richardson

**Affiliations:** 1 Institute of Biology, Leiden University, Sylvius Laboratory, Leiden, The Netherlands; 2 Department of Medical Pharmacology, Leiden/Amsterdam Center for Drug Research, Leiden, The Netherlands; 3 Institute of Chemistry, Leiden University, Leiden, The Netherlands; Ecole Normale Supérieure de Lyon, France

## Abstract

**Background:**

In humans, ethanol exposure during pregnancy causes a spectrum of developmental defects (fetal alcohol syndrome or FAS). Individuals vary in phenotypic expression. Zebrafish embryos develop FAS-like features after ethanol exposure. In this study, we ask whether stage-specific effects of ethanol can be identified in the zebrafish, and if so, whether they allow the pinpointing of sensitive developmental mechanisms. We have therefore conducted the first large-scale (>1500 embryos) analysis of acute, stage-specific drug effects on zebrafish development, with a large panel of readouts.

**Methodology/Principal Findings:**

Zebrafish embryos were raised in 96-well plates. Range-finding indicated that 10% ethanol for 1 h was suitable for an acute exposure regime. High-resolution magic-angle spinning proton magnetic resonance spectroscopy showed that this produced a transient pulse of 0.86% concentration of ethanol in the embryo within the chorion. Survivors at 5 days postfertilisation were analysed. Phenotypes ranged from normal (*resilient*) to severely malformed. Ethanol exposure at early stages caused high mortality (≥88%). At later stages of exposure, mortality declined and malformations developed. Pharyngeal arch hypoplasia and behavioral impairment were most common after *prim-6* and *prim-16* exposure. By contrast, microphthalmia and growth retardation were stage-independent.

**Conclusions:**

Our findings show that some ethanol effects are strongly stage-dependent. The phenotypes mimic key aspects of FAS including craniofacial abnormality, microphthalmia, growth retardation and behavioral impairment. We also identify a critical time window (*prim-6* and *prim-16*) for ethanol sensitivity. Finally, our identification of a wide phenotypic spectrum is reminiscent of human FAS, and may provide a useful model for studying disease resilience.

## Introduction

Alcohol (ethanol, ethyl alcohol) abuse resulted in economic costs to society of around US$148 billion in 1992 in the USA and resulted in 40,000 deaths [1]. One of the health consequences of alcohol is fetal alcohol syndrome (FAS), a condition in humans resulting from exposure of the developing embryo to ethanol [2]–[6]. The clinical features of FAS can be broadly divided into growth retardation, morphological malformations (especially craniofacial defects) and central nervous system impairment [7]–[9]. The craniofacial defects include eye abnormalities such microphthalmia [10], as well as various defects that have been interpreted as first or second pharyngeal arch abnormalities (e.g., hearing disorders and ear malformations [11], and thin upper lip). Individuals with all of these categories of defect are at the most severely affected end of a continuous spectrum of alcohol teratogenicity. While some offspring of mothers who drink heavily during pregnancy develop FAS with all the symptoms described above, some show no symptoms at all (a condition known as ethanol resilience [12], [13]) while many more show partial FAS-related phenotypes. For example, children mainly showing a range of impairments affecting intellectual functioning may be categorized under the term fetal alcohol spectrum disorder (FASD) [14], [15]. All together, these findings suggest that environmental and genetic factors from the fetal compartment may confer a certain degree of vulnerability or resilience to ethanol-induced teratogenesis and that certain tissues, organs or systems appear to be more vulnerable than others depending on dose, duration and timing of exposure to alcohol [9], [12].

A wide array of mammalian models has been used to examine the mechanisms underlying FAS-related phenotypes (reviewed in [16], [17]). Neural crest cells that populate the first and second pharyngeal arches and outflow tract of the heart, as well as neuronal and glial stem cells in the central nervous system are particularly affected by ethanol exposure (reviewed by [18]. An important but unresolved question is when exactly is the critical period(s) for ethanol exposure during embryogenesis and which of the molecular components expressed during such periods are ethanol-sensitive. This is difficult to establish precisely in mammalian embryos inside the womb, especially given variations within and among litters [19].

The zebrafish model resolves these staging issues, allowing the study of developmental processes in a non-invasive manner [20]–[22]. Owing to their transparency, development and internal processes of both embryos and larvae can be easily visualized microscopically, allowing real-time analysis. Furthermore, the embryos become motile at early developmental stages, allowing behavioral analyses to be made in very young animals in response to ethanol [23], [24].

Previous studies using zebrafish embryos have reported a range of effects of ethanol including developmental retardation, pericardial and yolk-sac oedema [9], [25], reduction in body length [26], branchial skeleton defects [27], abnormal eye development [10], [28]–[31] as well as cognitive defects [27], [32] and higher mortality [33]. Since this cluster of defects overlaps with human FAS, these findings support the view that zebrafish represents an ideal model to study ethanol effects.

To date, the majority of studies of ethanol toxicity in zebrafish have used chronic exposure, often over several hours or days (Table 1). This makes it difficult to identify critical developmental stages of sensitivity to ethanol. Because the zebrafish develops so rapidly, especially at the early stages, a short exposure time is required if the embryo is to remain at the same stage during the exposure. For this reason, we will here use a relatively brief pulse of ethanol exposure.

**Table 1:** Summary of selected literature on ethanol toxicity in zebrafish.

Duration of exposure	Stage of exposure	ethanol %	Assay	Readout(s)	Plate format	Ref.
Acute (1 h)	3–4 month	0.25–1.0	immediate	behavior	aquarium (15 L)	[52]
Acute (2 h)	1 dpf	0.25–1.0	delayed	behavior (6 month old)	Petri dish, 60 per dish or tank, 20 per tank	[15]
Acute (3 h)	256 cells, high, dome/30% epiboly, germ-ring*	2.4	delayed	eye morphology	Petri dishes or glass beakers	[36]
Acute (1 h)	4 month	0.25–1.0	immediate	behavior (adult)	tank	[70]
Acute (1 h)	6 dpf	1.0–4.0	immediate	behavior at 6 dpf	96-well plate	[24]
Acute (20 min)	7 dpf	0.5–4.0%	immediate	behavior and melanocytes	10 per chamber8×6×2 cm	[48]
Chronic	6–24, 12–24, 24–36, 48–60, 60–72 hpf	1.5	delayed	visual function between 3–9 dpf	Petri dish	[23]
Chronic (2 weeks)	Young adult	0.5	immediate	behavior	5 gallon aquarium	[71]
Chronic	6–24, 12–24, 24–36, 48–60, 60–72 hpf	1.5–2.9	delayed	eye diameter and physical abnormalities between 3–7 dpf	Petri dish	[31]
Chronic	1 dpf	4.0	delayed	*hsp47 and hsp70* gene expression (2 dpf)	aquarium	[72]
Chronic (3 days)	1 dpf	0.1–1.0	delayed	eye morphology	6-well plate, 10 per well	[29]
Chronic (3 days)	2 dpf	1.0–2.0	delayed	eye morphology	6-well plate	[30]
Acute (4 h)			4 h			
Chronic (6 h)	1 dpf	0.25–2.0	delayed	developmental defects (1–4 dpf)	Petri dish	[73]
Chronic (c. 20 h)	1 dpf	1.0–2.4	delayed	survival and eye morphology	Petri dish	[74]
Chronic (6 days)	1 dpf	0.02–1.9	immediate	neurobehavior and skeletal morphogenesis	24-well plate, 10 per well	[27]
Chronic (c. 20 h)	1 dpf	1.0–2.5	delayed	embryonic pattern formation and gene expression	5 ml (format not specified)	[26]
Chronic (c.20 h)	1 dpf	1.5–2.5	delayed	eye morphology (1–5 dpf)	Petri dishes or glass beakers	[75]
Chronic (c.24 h)	1 dpf	1.0–1.5	delayed	eye morphology	glass beaker	[28]

The table is intended to show the diversity of exposure and assay protocols used in this field. Note also the lack of published stage-specific acute treatments. Key:

*, stages according to [34].

We chose eight morphological stages covering the major phases of early development, namely the blastula period (*dome*), gastrulation (*50% epiboly*, *75% epiboly*), organogenesis and segmentation (*26- somite, prim-6, and prim-16*) and some later phases of organogenesis and tissue differentiation (*high pec*, *long pec*) [34].

## Materials and Methods

### Ethics statement

All animal experimental procedures were conducted in accordance with local and international regulations. The local regulation is the *Wet op de dierproeven* (Article 9) of Dutch Law (National) and the same law administered by the Bureau of Animal Experiment Licensing, Leiden University (Local). This local regulation serves as the implementation of *Guidelines on the protection of experimental animals* by the Council of Europe, Directive 86/609/EEC, which allows zebrafish embryos to be used up to the moment of free-living (approximately 5–7 days after fertilisation). Because embryos used here were no more than 5 days old, no licence is required by Council of Europe (1986), Directive 86/609/EEC or the Leiden University ethics committee.

### Animals

Male and female adult zebrafish (*Danio rerio*) of AB wild type were purchased from Selecta Aquarium Speciaalzaak (Leiden, the Netherlands) who obtain stock from Europet Bernina International BV (Gemert-Bakel, the Netherlands). Fish were kept at a maximum density of 100 individuals in glass recirculation aquaria (L 80 cm; H 50 cm; W 46 cm) on a 14 h light: 10 h dark cycle (lights on at 8 h). Water and air were temperature controlled (25±0.5°C and 23°C, respectively). All animal handling was in accordance with local and national regulations. The fish were fed twice daily with ‘Sprirulina’ brand flake food (O.S.L. Marine Lab., Inc., Burlingame, USA) and twice a week with frozen food (Dutch Select Food, Aquadistri BV, the Netherlands).

### Embryo buffer

To produce a defined and standardized vehicle (control) for these experiments, we used 10% Hank's balanced salt solution (made from cell-culture tested, powdered Hank's salts, without sodium bicarbonate, Cat. No H6136-10X1L, Sigma-Aldrich, St Louis, MO) at a concentration of 0.98 g/L in Milli·Q water (resistivity = 18.2 MΩ·cm), with the addition of sodium bicarbonate at 0.035 g/L (Cell culture tested, Sigma Cat S5761), and adjusted to pH 7.46. A similar medium was previously used [24], [35].

### Egg water

Egg water was made from 0.21 g ‘Instant Ocean®’ salt in 1 L of Milli-Q water with resistivity of 18.2 MΩ·cm.

### Embryo care

Eggs were obtained by random pairwise mating of zebrafish. Three adult males and four females were placed together in small breeding tanks (Ehret GmbH, Emmendingen, Germany) the evening before eggs were required. The breeding tanks (L 26 cm; H 12.5 cm; W 20 cm) had mesh egg traps to prevent the eggs from being eaten. The eggs were harvested the following morning and transferred into 92 mm plastic Petri dishes (50 eggs per dish) containing 40 ml fresh embryo buffer. Eggs were washed four times to remove debris, while unfertilized, unhealthy and dead embryos were removed under a dissecting microscope. At 3.5 hpf, embryos were again screened and any further dead and unhealthy embryos were removed. Throughout all procedures, the embryos and the solutions were kept at 28.5°C, either in the incubator or a climatised room. All incubations of embryos were carried out in an incubator with orbital shaking (50 rpm) under a light cycle of 14 h light: 10 h dark (lights on at 8 h). Embryo buffer was refreshed every 24 h. All pipetting was done manually, with an 8-channel pipetter.

### Acute ethanol exposure

When the embryos in the Petri dishes had reached the required developmental stages [34], they were gently transferred using a sterile plastic pipette into 96-well microtitre plates (Costar 3599, Corning Inc., NY) at a density of one embryo per well. A single embryo was plated per well. We used this plating density for two reasons: first, so that embryos that subsequently died would not affect the others; and second, to allow individual embryos to be tracked for the whole duration of the experiment, including recording of the behavior of individual embryos.

Each well contained 250 µL of either 10% (1.64 M) ethanol in embryo buffer, or buffer only (which we refer to as control or vehicle). The ethanol was high purity, medical grade (‘Emprove’ ethanol, Cat. No. 100971, Merck KGaA, Darmstadt, Germany). To minimize handling stress, embryos were not dechorionated because previous reports suggested the chorion to be freely permeable to ethanol [36].

### Range-finding

We conducted range-finding to identify a suitable effective ethanol concentration. For this we used 1 h acute exposure of 0, 2, 4, 8, 16 and 32% ethanol at *75% epiboly*, *26-somite*, *prim-16* and *long pec* stages. We used 32 embryos for each concentration at all stages of exposure. At 5 dpf, mortality was recorded and LC_50_ (Table 2) was calculated using the Probit analysis function of SPSS Statistics (version 17.0).

**Table 2:** LC_50_ of ethanol (1 h exposure), at different developmental stages, and recorded at different timepoints (hpf).

	LC_50_ (% ethanol) recorded at following timepoints			
stage of 1 h ethanol exposure:	48 hpf	72 hpf	96 hpf	120 hpf
*75% epiboly*	5.5	5.5	5.5	5.5
*26-somite*	10.93	10.6	10.6	10.6
*prim-16*	9.77	9.77	9.77	9.53
*long pec.*	n.a.	9.33	9.33	9.33

We used 0, 2, 4, 8 and 16% ethanol.

Key: n.a., not applicable.

### Ethanol treatment

For each stage, we used 48 embryos for ethanol treatment and 48 embryos for control in alternating columns of 8 wells within the 96-well plate. For ethanol treatment, an acute 1 h exposure was used. This was followed by 3–4 washes with fresh embryo buffer. Embryos were kept in an incubator at 28.5°C, with refreshment of the buffer once daily, until 5 dpf according to the following procedure: for each fluid renewal, 175 µL was first withdrawn from the total of 250 µL in the well in order to leave the embryo completely covered by the residual volume (75 µL) of buffer. Then, 175 µL of fresh buffer was added to each well.

### Determination of ethanol concentration in embryos by high-resolution magic-angle spinning proton magnetic resonance spectroscopy (HR-MAS ^1^H MRS)

Zebrafish embryos with intact chorions at *prim-6* were divided into the following treatment groups: (i) 10% ethanol for 1 h (ii) vehicle only for 1 h (iii) 10% ethanol for 1 h followed by three washes with fresh buffer (iv) 10% ethanol for 1 h followed by three washes with fresh buffer and further incubation for 1 h, 4 h or 24 h in buffer. All samples were then briefly drained and then frozen at −80°C. For HR-MAS ^1^H MRS measurement, intact embryos were placed in a 4 mm Bruker zirconium rotor and subsequently 50 µL of 100 mM deuterated phosphate buffer (pH 7.4) containing 3-trimetylsilyl-2,2,3,3-tetradeuteropropionic acid (1 mM TSP) was added. The rotor was immediately placed in a Bruker Avance 400 spectrometer. The whole HR-MAS study was performed at 4°C to minimize tissue degradation. The spectra were acquired at a spinning rate of 2500 rpm using a Carr-Purcell-Meiboom-Gill pulse sequence with the repetition time and echo time of 3500 ms and 0.4 ms respectively. The concentration of ethanol in the embryos was determined by comparing the integral peak intensity of the CH_3_ and CH_2_ protons of ethanol with that of the TSP peak, after correcting for the number of contributing protons and for embryo weight. Furthermore, the concentration of total creatine inside embryos was used as internal reference to confirm the quantification of ethanol concentration (Table 3).

**Table 3:** Internal concentration of ethanol in intact embryos measured by high-resolution magic-angle spinning proton magnetic resonance spectroscopy (HR-MAS ^1^H MRS).

	Sample	Ethanol level inside the embryos (%)
1	Control (treated with buffer only)	0
2	Embryos treated with 10% ethanol for 1 h (without subsequent washing)	0.86
3	Embryos treated with 10% ethanol for 1 h and then washed 3× with buffer	0.0003
4	Embryos treated with 10% ethanol for 1 h, washed 3× with buffer and then allowed to grow for another 1 h	0
5	Embryos treated with 10% ethanol for 1 h and then washed 3× with washing buffer and then allowed to grow for another 3 h	0
6	Embryos treated with 10% ethanol for 1 h and then washed 3× with washing buffer and then allowed to grow for another 24 h	0
7	Positive control (embryos mixed with an equal volume of 10% ethanol)	5%

### Behavioral analysis

At 5 dpf, all living embryos were subjected to the *light/dark challenge* test. We were unable to exclude embryos with morphological abnormalities because such embryos could only be identified later, after fixation and staining. The light/dark challenge test consists of brief (less than 10 min) frequently alternating periods of light and dark. We chose four minute sessions to prevent habituation, and also to favor more robust behavioral changes. The test procedure produces robust changes in locomotor activity in larval zebrafish as young as 5 dpf, and can be easily performed in a 96-well plate. Typical behavioral responses include low (basal) locomotor activity under light exposure followed by robust behavioral hyperactivity upon sudden transition to dark. Locomotor activity levels are readily restored to that of basal values upon rapid re-exposure to light [24], [35]. This pattern of response is observed because sudden changes in illumination can temporarily override activity levels set by the circadian clock, an effect similar to masking in higher vertebrates [37], [38]. Such ability to detect changes in illumination (if not due to nightfall) is believed to have evolved to encourage animals to seek bright environments, where feeding and predator avoidance can be better optimized than in dark zones [24], [39], [40].

Because of the robustness of the behavioral changes induced by varying illumination, this task can be used to reveal more readily than any other tasks, defective brain function, aberrant nervous system development and/or locomotor and visual defects caused by teratogenic agents such as ethanol.

Live embryos were analyzed in the ZebraBox recording apparatus with VideoTrack software (both from Viewpoint S.A., Lyon, France). Their swimming patterns and other movements were recorded automatically according to the following sequence: the locomotor activity was recorded for a period of 14 min, which was further divided into 4 blocks. Block 1: lights ON for 2 min (pre-test adaptation period); block 2: lights ON for 4 min (measures basal activity); block 3: Lights OFF for 4 min (measures responsiveness to a sudden pulse of darkness); and block 4: Lights ON for 4 min (measures recovery from darkness pulse). Alterations in locomotor activity in any of these blocks can be used to provide an index of physiological alterations (either in terms of locomotor or visual impairment). After the recording, the experiment was terminated and all embryos were processed for morphological assessment.

### Morphometric analysis

Digital images were made of the dorsal aspect of surviving embryos, after fixing, staining and clearing in glycerol (see above). The images were captured using a Nikon SMZ-800 stereomicroscope fitted with a Nikon DS Fi1 digital camera. We calibrated and took measurements from the images using *Image J* (version 1.40, National Institutes of Health, MD). Two measurements were made: (i) *body length* (Figure S1 A), the distance from the tip of Meckel's cartilage to the tip of the tail; and (ii) *eye size* (Figure S1 B), the longitudinal diameter of the left and right eyes (averaged per embryo).

### Morphological assessment of embryo phenotypes in the survivor population

Embryos were fixed in 4% paraformaldehyde (PFA) in phosphate-buffered saline at pH 7.2 at 4°C overnight. They were then rinsed 5 times in distilled water and dehydrated in a graded series of ethanol (25, 50, and 70%) for 5 min each. Embryos were rinsed in acid alcohol (1% concentrated hydrochloric acid in 70% ethanol) for 10 min. They were then placed in filtered Alcian blue solution (0.03% Alcian blue in acid alcohol) overnight. Embryos were subsequently differentiated in acid alcohol for 1 h and washed 2×30 min in distilled water. For photography, embryos were bleached as follows: they were placed in 0.05% trypsin (Type IIS porcine pancreas, Sigma Cat. No. T-7409) dissolved in a saturated solution of sodium tetraborate for 3 h, then bleached in a mixture of 3% hydrogen peroxide and 1% potassium hydroxide for 4 h. Finally, they were cleared and stored in glycerol. Care was taken not to overbleach, because this caused the tissue to disintegrate. All embryos remained in their original multiwell plates, so that each individual could be tracked throughout the entire experimental and analysis procedure. Analysis of embryo morphology was carried out using a dissecting stereo microscope. General outcomes of morphological analyses of embryos are summarized in Table 4. The phenotypes were scored according to the criteria listed in Table 5.

**Table 4:** General outcomes per stage of treatment.

						Morphology (5 dpf)¶		Severity of abnormality at 5 d‡		
		Total	Dead	Lost**	Survivors (5 dpf)	Normal	Abnormal	Mild	Moderate	Severe
Stage*	Treatment	N	N (%)	N (%)	N (%)	N (%)	N (%)	N (%)	N (%)	N (%)
***dome***	vehicle	48	7 (14.6)	18 (37.5)	23 (47.9)	n.a	n.a	n.a	n.a	n.a
	ethanol	48	39 (81.3)	9 (18.7)	0	n.a	n.a	n.a	n.a	n.a
***50% epiboly***	vehicle	48	1 (2.1)	15 (31.3)	32 (66.7)	n.a	n.a	n.a	n.a	n.a
	ethanol	48	44 (91.7)	4 (8.3)	0	n.a	n.a	n.a	n.a	n.a
***75% epiboly***	vehicle	48	1 (2.1)	20 (41.7)	27 (56.3)	n.a	n.a	n.a	n.a	n.a
	ethanol	48	46 (95.3)	2 (4.2)	0	n.a	n.a	n.a	n.a	n.a
***26 -somite***	vehicle	48	2 (4.2)	9 (18.7)	37 (77.1)	27 (73.0)	10 (27.0)	7 (70.0)	3 (30.0)	0
	ethanol	48	5 (10.4)	4 (8.3)	39 (81.3)	16 (41.0)	23 (59.0)	12 (52.2)	8 (34.8)	3 (13.0)
***prim-6***	vehicle	48	0	11 (22.9)	37 (77.1)	30 (81.1)	7 (18.9)	6 (85.7)	114.3)	0
	ethanol	48	13 (27.1)	7 (14.6)	28 (58.3)	8 (28.6)	20 (71.4)	12 (60.0)	2 (10.0)	6 (30.0)
***prim-16***	vehicle	48	5 (10.4)	11 (22.9)	32 (66.7)	20 (62.5)	12 (37.5)	9 (75.0)	3 (25.0)	0
	ethanol	48	12 (25.0)	10 (20.8)	26 (54.2)	4 (15.4)	22 (84.6)	13 (59.1)	3 (13.6)	6 (27.3)
***high pec***	vehicle	48	5 (10.4)	14 (29.2)	29 (60.0)	19 (65.5)	10 (34.5)	5 (50.0)	3 (30.0)	2 (20.0)
	ethanol	48	20 (41.7	12 (25.0)	16 (33.3)	6 (37.5)	10 (62.5)	4 (40.0)	0	6 (60.0)
***long pec***	vehicle	48	0	20 (41.7)	28 (58.3)	28 (100)	0	0	0	0
	ethanol	48	4 (8.3)	16 (33.3)	28 (58.3)	20 (71.4)	8 (28.6)	2 (25.0)	3 (37.5)	3 (37.5)
**Total**	vehicle	384	21 (6.0)	118 (30.7)	245 (63.8)	204 (83.3)	41 (16.7)	29 (70.7)	10 (24.4)	2 (4.9)
	ethanol	384	183 (47.7)	64 (16.7)	137 (35.7)	54 (39.4)	83 (60.6)	43 (51.8)	16 (19.3)	24 (28.9)

Overview of total number embryos treated, survival at 5 dpf, the presence of morphological abnormalities at 5 dpf, and the degree of severity of those abnormalities.

Key: n.a., not applicable;

*developmental stage [34] at which embryo was exposed to 10% ethanol (or vehicle only) for 1 h;

¶, morphology at 5 dpf was classified as normal or abnormal according to the criteria in Table 5; for selected illustrations of these phenotypes see Figure 3. The abnormal embryos were further subdivided into three categories of severity (‡) of the abnormality: mild, moderate or severe, according to the criteria listed in Table 6;

**‘Lost’ indicates that embryos were lost during processing (mostly through aspiration during pipetting of buffer or other reagents). Note that 23.7% of all embryos (ethanol and vehicle) were lost by 5 dpf. Very few embryos survived after treatment at the earliest three stages (*dome*, *50% epiboly* and *75% epiboly*) with ethanol but all lost. For these reasons, these stages are not analyzed further.

**Table 5:** Phenotype analysis.

Larval phenotype	Criteria
*1. Normal*	Absence of any of the phenotypes listed below
*2. Eye*	Presence of gross microphthalmia in one or both eyes
*3. Heart*	Presence of pericardial oedema
*4. Yolk*	Presence of yolk sac oedema
*5. Meckel's cartilage*	Meckel's cartilage grossly hypoplastic, missing or unfused in midline. These effects may be unilateral or bilateral.
*6. Branchial arches*	One or more cartilages of the branchial skeleton hypoplastic or missing.
*7. Pectoral fins*	One or both pectoral fins hypoplastic or missing.

Description of the seven categories used to score larval phenotype at 5 dpf. See Figure 3 for selected illustrations of these phenotypes.

### Severity of morphological effect per embryo

In addition to recording the frequency in the survivor population of different morphological phenotype categories (Table 5) we further analyzed the extent to which individual embryos were abnormal. We expressed this individual burden of phenotypic abnormalities in terms of a severity scale see Table 6. Please note that the determination of severity is to some extent subjective.

**Table 6:** Phenotypic variation analysis.

Severity	Criteria
*Mild*	An individual embryo had any **one of any** type of defect from 2–7, in Table 5.
2–7, in Table 5.	
*Moderate*	An individual embryo had a minimum of any **two non-branchial**, **non-Meckel's cartilage** abnormalities; i.e. the embryo showed two from categories 2–4, or 7, in Table 5.
*Severe*	An individual embryo had abnormality of the **branchial arches** and/or **Meckel's cartilage** combined with **at least one** other of defects 2–7 in Table 5.

Severity scale used to express the degree to which individual embryos were phenotypically abnormal. See Figure 3 for selected illustrations of these phenotypes.

### Statistical analysis

Statistical analyses were performed using SPSS for Windows (version 12.0.1). Graphs were plotted using Prism Graph Pad software (5.03). Chi-square (student exact) test was employed for survival rate. Quantitative morphological analyses for body length and eye size were performed using unpaired (two-tailed) student's *t* test. Two-way ANOVA for repeated measurements with treatment (vehicle and ethanol) as a between-subjects factor and behavioral phases (basal, challenge, and recovery) as a within-subjects factor was used to analyze total distance swum, as well as percentage of time swimming with high velocity, in response to the light/dark challenge test. Mauchly's test of sphericity was applied and the degrees of freedom (df) corrected to more conservative values using the Huynh–Feldt (H-F) if the assumption of sphericity was violated. Significant main effects were further decomposed using pairwise comparisons with a Bonferroni's correction, for multiple comparisons. Data are presented as mean ±SEM, and a probability level of 5% was used as the minimal criterion of significance.

## Results

### General findings

**Figure 1:**
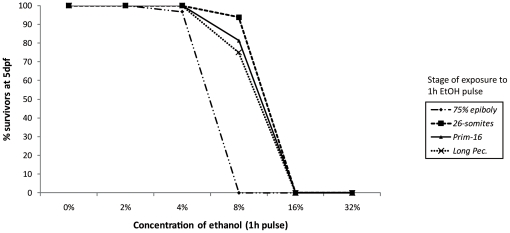
Survival with a geometric series of ethanol concentrations (1 h exposure), at various developmental stages. The ethanol concentrations used were: 0, 2, 4, 8, 16 and 32%, Mortality was recorded at various intervals after exposure (48, 72, 96 and 120 hpf).

We performed preliminary range-finding experiments with 2, 4, 8 and 16% ethanol exposures for 1 h. These showed the LC_50_ for ethanol to be between 9.33 and 10.93% at *26-somite* to *long pec* stages (Table 2, Figure 1). For the sake of standardisation, we used 10% ethanol for 1 h in all subsequent experiments.

### Ethanol concentration in treated embryos

**Figure 2:**
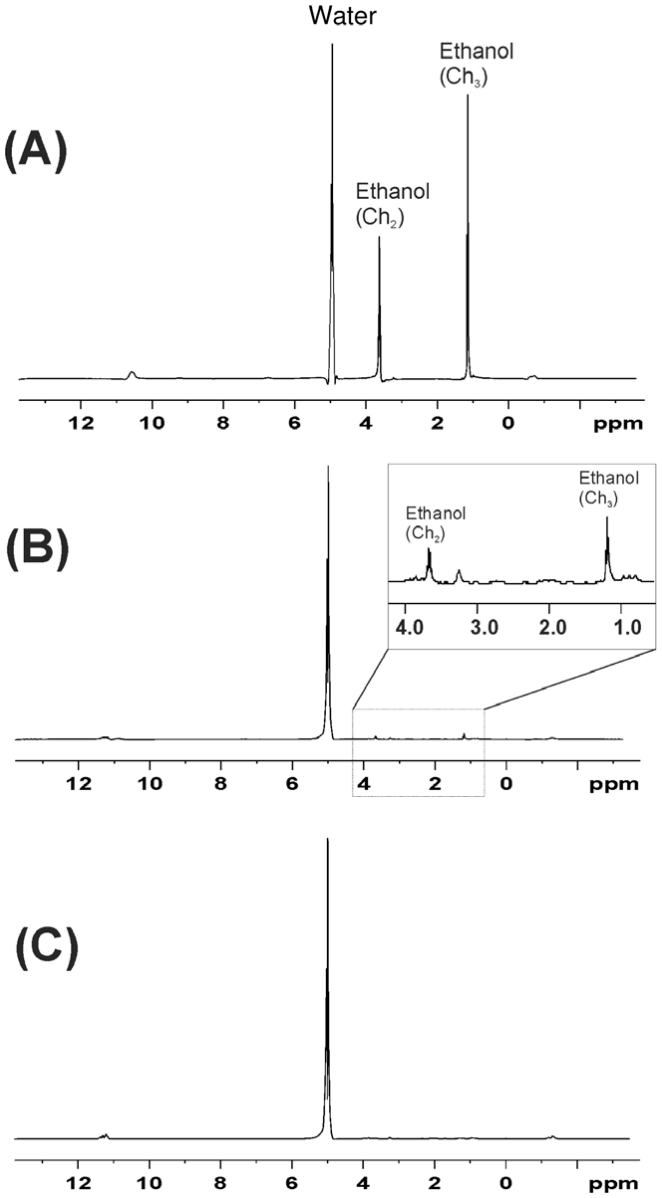
Ethanol concentration in treated embryos. Embryos with chorion were treated with 10% ethanol for 1 h and the HR-MAS ^1^H MRS spectra recorded. **A**, without subsequent washing; **B**, after washing three times with buffer. The inset shows a detail enlarged 30 times with respect to the *y*-axis; **C**, after washing three times with buffer and subsequently allowed to grow for another 1 h.

Results of high-resolution magic-angle spinning proton MRS (HR-MAS ^1^H MRS) in intact embryos are shown in Figure 2 and Table 3. At the end of the 1 h ethanol treatment, but before rinsing in buffer, the ethanol level in the embryos had risen to 0.86%. After 3× rinsing with buffer, the ethanol concentration in the embryos had fallen to 0.0003%.

### Ethanol-induced lethality and incidence of malformations by stage of exposure

Few survivors were obtained after treatment of the earliest three stages (*dome*, *50% epiboly* and *75% epiboly*) with ethanol. For these reasons, these stages are not analyzed further. By contrast, 87.5%, 98% and 98%, respectively, of embryos treated at these stages with vehicle survived. Thus the mortality rates are significantly higher with ethanol treatment (Chi-square, Fisher's exact test, all *p*s<0.01). Mortality after exposure to ethanol (Figure S2) drops dramatically from *26-somites* stage onwards, with only 8.3% mortality at the last stage of exposure examined (*long pec*). In Figure S3, it can be seen that the incidence of morphologically abnormal embryos among survivors is consistently higher in the ethanol-treated group than in the controls. Furthermore, among the ethanol-treated populations, the percentage of morphologically abnormal embryos is highest after treatment at *prim-16* (84.6%). Note that there is a low level of morphologically abnormal embryos (mild pericardial and yolk sac oedemas only) that occurs among the vehicle population.

### Ethanol exposure during specific stages of embryogenesis causes craniofacial alterations that vary in degrees of severity

**Figure 3:**
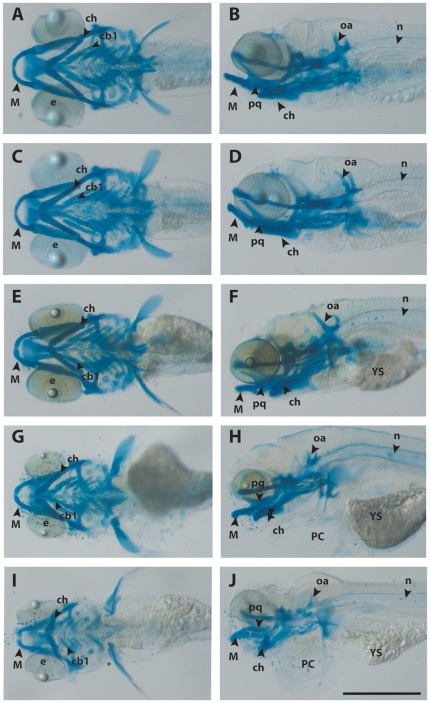
Morphological analysis reveals the degree of severity of malformations. Zebrafish embryos at 5 dpf stained with Alcian blue to show cartilage of the head and branchial region. The aim of this figure is to show examples of the range of severities of malformation obtained (Table 6). **A**, **C**, **E**, **G**, **I**, ventral views; **B**, **D**, **F**, **H**, **J**, left lateral views. In all figures, rostral is to the left. All embryos are shown to the same scale, indicated by the scale bar (500 µm in **J**). All embryos were exposed at *prim-16* to either vehicle alone (**A**, **B**) or 10% ethanol (**C**–**J**). **A**, **B**, vehicle only, embryo classified as ‘normal’. **C**, **D**, ethanol-treated, embryo classified as ‘normal’. **E**, **F**, ethanol-treated embryo classified as ‘mild’. The embryo shows yolk sac oedema. **G**, **H**, ethanol-treated embryo classified as ‘moderate’. The embryo shows oedema of the yolk sac and pericardium as well as gross microphthalmia. **I**, **J**, ethanol-treated embryo, phenotype classified as ‘severe’. The embryo shows gross microphthalmia, pericardial and yolk sac oedema, and grossly hypoplastic Meckel's and branchial cartilages. Key: *cb1*, 1^st^ ceratobranchial cartilage; *ch*, ceratohyal cartilage; *e*, eye; *M*, Meckel's cartilage; *n*, notochord; *oa*, occipital arches; *pc*, pericardium and heart; *pq*, palatoquadrate; *ys*, yolk sac.

**Figure 4:**
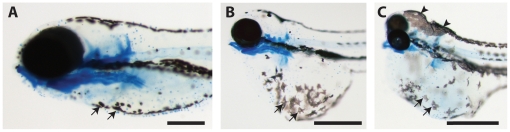
Morphology of melanocytes at 5 dpf in embryos treated with ethanol. All embryos were fixed, stained with Alcian blue and cleared in glycerol. **A**, embryo treated at *long-pec* with vehicle only and having a normal phenotype. Note that the melanocytes on the ventral body (arrows) are contracted and punctuate in appearance (scale bar = 250 µm). **B**, **C**, embryos treated at *high-pec* with ethanol and having severe phenotypes (scale bars = 500 µm); note that the melanocytes on the yolk sac (arrows) have a dispersed morphology; in **C**, the melanocytes on the dorsal surface of the head are also dispersed and form a pavemented layer (arrowheads).

**Figure 5:**
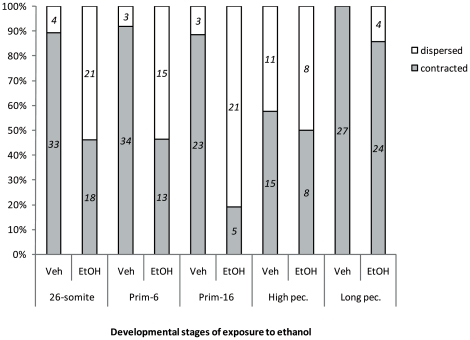
Quantification of melanocyte phenotype at 5 dpf in embryos treated with ethanol at different stages. ‘Contracted morphology’ indicates that the cell is rounded, and the melanosomes concentrated into a small area (Figure 4 A). ‘Dispersed’ morphology (Figure 4 B,C) indicates that the yolk sac melanocytes were squamate and the melanosomes distributed across a wider area than in Figure 4A. As can be seen in the graph, the dispersed morphology is characteristic of ethanol-treated embryos, and reaches a maximum in embryos treated at *prim-16*. Italic numbers = N embryos.

Results of morphological analyses of embryos are summarized in Table 4. The wide range of phenotypic effects that can be seen in one treatment group is illustrated in Figure 3 which compares an untreated embryo (Figure 3A,B) with embryos exposed to ethanol at *prim-16* (Figure 3C–J). One subpopulation in this treatment group appears normal (Figure 3C–D). The embryo in Figure 3E–F illustrated a ‘mild’ malformation phenotype, in this case, yolk sac oedema, but no other gross malformations. A ‘moderate’ malformation phenotype is illustrated by the embryo in Figure 3G–H which shows yolk sac oedema, pericardial oedema, microphthalmia and hypoplasia of Meckel's cartilage. The embryo in Figure 3I–J shows ‘severe’ malformations, including severe microphthalmia, Meckel's hypoplasia, branchial arch cartilage hypoplasia, pericardial oedema and yolk sac oedema. The effects on melanocyte morphology depended on stage of treatment. As can be seen in (Figure 4 and Figure 5), the ‘dispersed’ morphology, characteristic of ethanol-treated embryos, is most prevalent in embryos treated at *prim-16*. Note that we did not look at iridophores or xanthophores.

**Figure 6:**
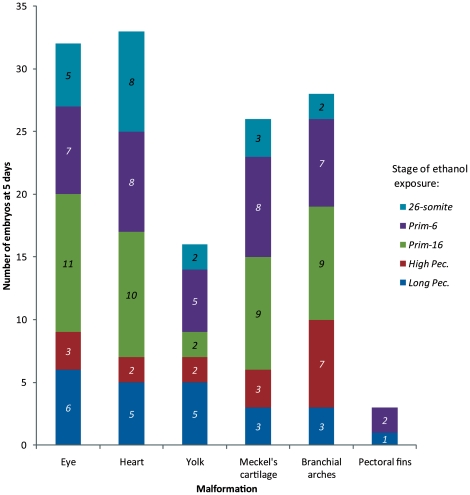
Stage-dependent sensitivity of the different anatomical regions. Note that eye development is sensitive to ethanol exposure at all developmental stages (but most sensitive at *prim-16*). Meckel's cartilage was particularly sensitive to ethanol exposure at *prim-6* and *prim-16*. The branchial arches were most sensitive to ethanol exposure at *prim-6*, *prim-16*, and *high pec*. In contrast to these stage-specific effects, the presence of oedema (i.e. the ‘heart’ and ‘yolk’ categories) was present at low levels following exposure at all stages. Italic numbers = N embryos.

**Figure 7 pone-0020037-g007:**
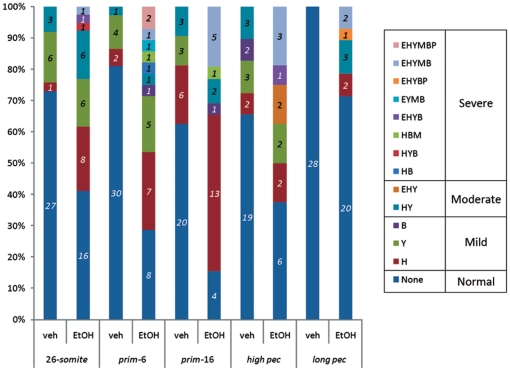
Clustering of morphological abnormalities per embryo. Number on bars indicates the number of embryos with a particular combination of defects, or single defect, or no gross defect (normal). Surviving embryos were classified according to their phenotype. Key: Normal, no abnormalities; H), embryos with pericardial oedema only; Y, embryos with yolk sac oedema only; B, embryos with branchial arch abnormalities only; HY, embryos with pericardial and yolk sac oedema only; EHY, embryos with microphthalmia, pericardial and yolk sac oedema only; HB, embryos with pericardial oedema and branchial abnormalities only; HYB, embryos with pericardial oedema, yolk sac oedemas and branchial arch abnormalities only; HBM, embryos with pericardial oedema, branchial arch and Meckel's cartilage malformations only; EHYB, embryos with microphthalmia, pericardial oedema, yolk sac oedema and branchial arch defects only; EYMB, embryos with microphthalmia, yolk sac oedema, Meckel's cartilage and branchial arch defects only; EHYBP, embryos with microphthalmia, pericardial oedema, yolk sac oedema, branchial arch and pectoral fin abnormalities only; EHYMB, embryos with microphthalmia, pericardial oedema, yolk sac oedema, Meckel's cartilage and branchial arch abnormalities only; EHYMBP, embryos with microphthalmia, pericardial oedema, yolk sac oedema, Meckel's cartilage, branchial arch and pectoral fin abnormalities only. Italic numbers = N embryos.

We next analyzed the extent to which different malformations were associated with ethanol treatment at particular stages (Figure 6). We analysed the data using a generalized linear model of a Poisson model on a contingency table. We compared the levels with *high-pec* because it had the lowest counts. The results are shown in Table S1. There were significantly more incidences of malformations after *prim-6* and *prim-16* exposure. Varying the stage of exposure had no significant effect on the type of malformation (Figure 7).

**Figure 8:**
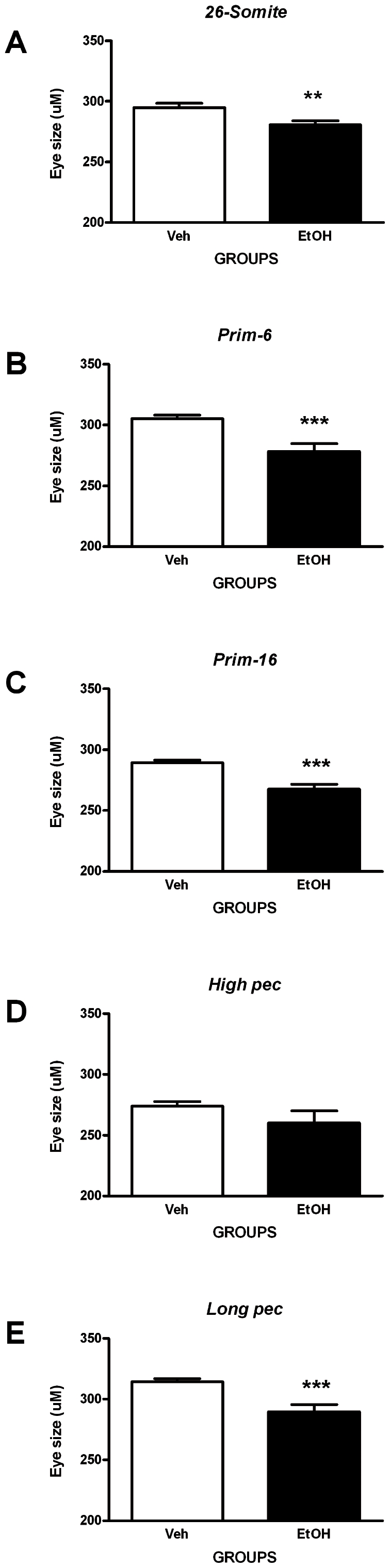
Assessment of microphthalmia-like phenotype. Ethanol treatment is associated with microphthalmia (assayed by measuring eye size at 5 dpf; see Figure S1 B). The graphs show eye size data (µm) for embryos exposed to an acute pulse of 10% ethanol (or vehicle only), for 1 h, at different developmental stages as follows: **A**, *26-somites*; **B**, *prim-6*; **C**; *prim-16*; **D**, *high-pec*; **E**, *long-pec*; stages. Statistical analysis (see methods) shows that ethanol exposure at all of these stages except *high pec* produced significant reduction in eye size (microphthalmia); this effect appears particularly pronounced after exposure at the *prim-6*, *prim-16* and *long pec* stages. Each error bar represents ±SEM of N = 37, 37, 32, 29, 27 embryos for vehicle and 39, 28, 26, 16, 28 for ethanol treatment at *26-somite*, *prim-6*, *prim-16*, *high pec*, and *long pec* respectively. Statistical icons: ** = p<0.01, and *** = p<0.001.

Eye development was found to be sensitive to ethanol exposure at all developmental stages, and the results were significant (see, Figure 8), with the largest incidence of eye abnormalities scored during the *prim-16* stage. A further statistical analysis was done by.

The clustering of malformations per embryo is shown in (Figure 7). The following observations can be made. Only two embryos out of 167 control embryos had any morphological defect (in this case, mild Meckel's cartilage hypoplasia). The percentage of embryos possessing one or more abnormality is maximum in the *prim-6* and *Prim-16* ethanol-exposed embryos; exposure to ethanol at earlier or later stages than these results in a decrease in the percentage of abnormal embryos. *Prim-6* and *Prim-16* ethanol treatment also led to the highest incidence of multiple organ abnormalities per embryo (i.e. abnormalities excluding oedema). There is a decrease in the percentage of ethanol-treated embryos showing oedema alone, as the stage of treatment increases.

### Ethanol exposure during embryogenesis causes microphthalmia-like phenotype and growth retardation in surviving larvae

#### Microphthalmia-like phenotype

Compared to vehicle-treated embryos, we find a significant reduction in the size of the eyes of ethanol-treated embryos at the following stages: *26-somite*, *prim-6*, *prim-16* and *long pec*. No differences in eye size were observed in embryos treated with ethanol at *high pec*. These findings are summarized graphically in Figures 6–8.

#### Growth retardation

**Figure 9:**
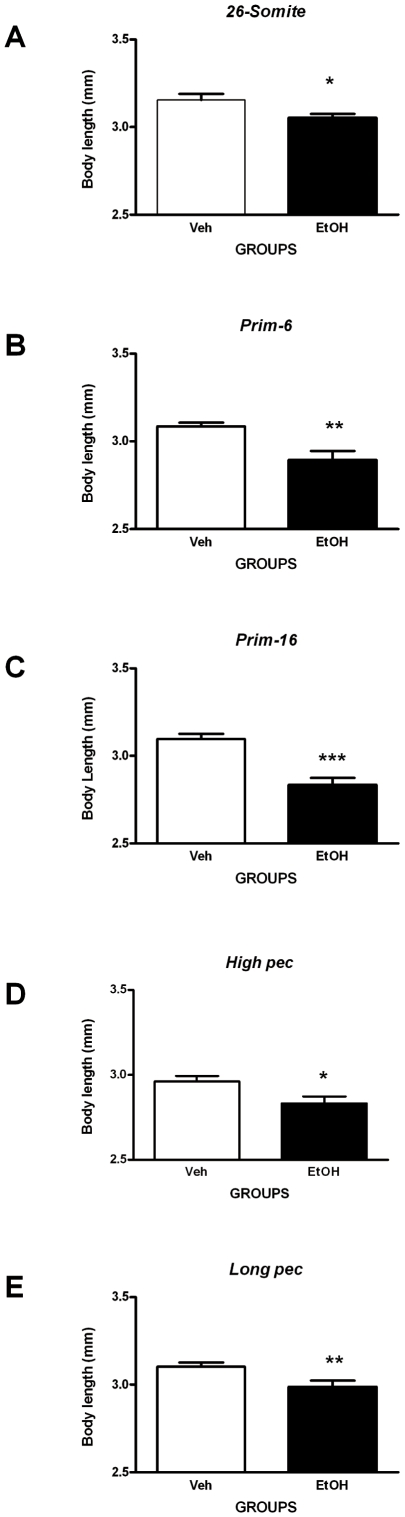
Assessment of skeletal growth. Ethanol treatment can produce growth retardation in zebrafish embryos (assayed by measuring body length at 5 dpf; see Figure S1 A). The graphs show body length data (mm) for embryos exposed to an acute pulse of 10% ethanol (or vehicle only), for 1 h, at different developmental stages as follows: **A**, *26-somites*; **B**, *prim-6*; **C**; *prim-16*; **D**, *high-pec*; **E**, *long-pec*. Statistical analysis shows that ethanol exposure at all 5 of these stages produced significant growth retardation; this effect was most striking after exposure at the *prim-16* stage. Each error bar represents ±SEM of N = 37, 37, 32, 29, 27 embryos for vehicle and 39, 28, 26, 16, 28 for ethanol treatment at *26-somite*, *prim-6*, *prim-16*, *high pec*, and *long pec* respectively. Statistical icons: * = p<0.05, ** = p<0.01, and *** = p<0.001.

We find a pervasive and significant reduction in body length in ethanol-treated compared to vehicle-treated embryos at all developmental stages studied from *26-somite* to *long pec* inclusive (Figure 9).

### Ethanol treatment causes a slight developmental delay

All batches of ethanol treated embryos, when analysed at 5 dpf, showed a delay in the development of selected staging criteria (data not shown) compared to controls. For example, in the ethanol-treated populations, the swim bladder was inflated in 121/137 (88.3%) surviving embryos while in the vehicles it was inflated in 166/167 (99.4%). Since swim bladder inflation is a staging criterion [34], this could indicate that ethanol treatment delays development.

### Ethanol exposure during critical periods of embryonic development causes lasting alterations in locomotor function

We next sought to determine the impact of microphthalmia-like phenotype and skeletal growth retardation on locomotor function using a behavioral test relying on the integrity of both eye and locomotor/skeletal system development, the light/dark challenge test. We first tested whether all larvae included in our analyses were apt to perform the behavioral test as expected (i.e. respond to sudden change in lighting conditions with alterations in swimming behavior). Statistical analyses confirm that this is indeed the case. Thus, for all developmental stages studied, a simple main effect of PHASE was observed [Fs_(2.0)_≥12.505, all *p*s<0.001]. These findings indicate that, in general, all larvae regardless of treatment (vehicle or ethanol) displayed a significant increase in locomotor activity (total distance moved) in the challenge phase (block 3, lights off) of the behavioral task when compared to the basal phase (block 2, lights on). Furthermore, levels of locomotor activity were found to rapidly return to values comparable to those observed in the basal phase when lights were turned on again in the recovery phase (block 4).

**Figure 10:**
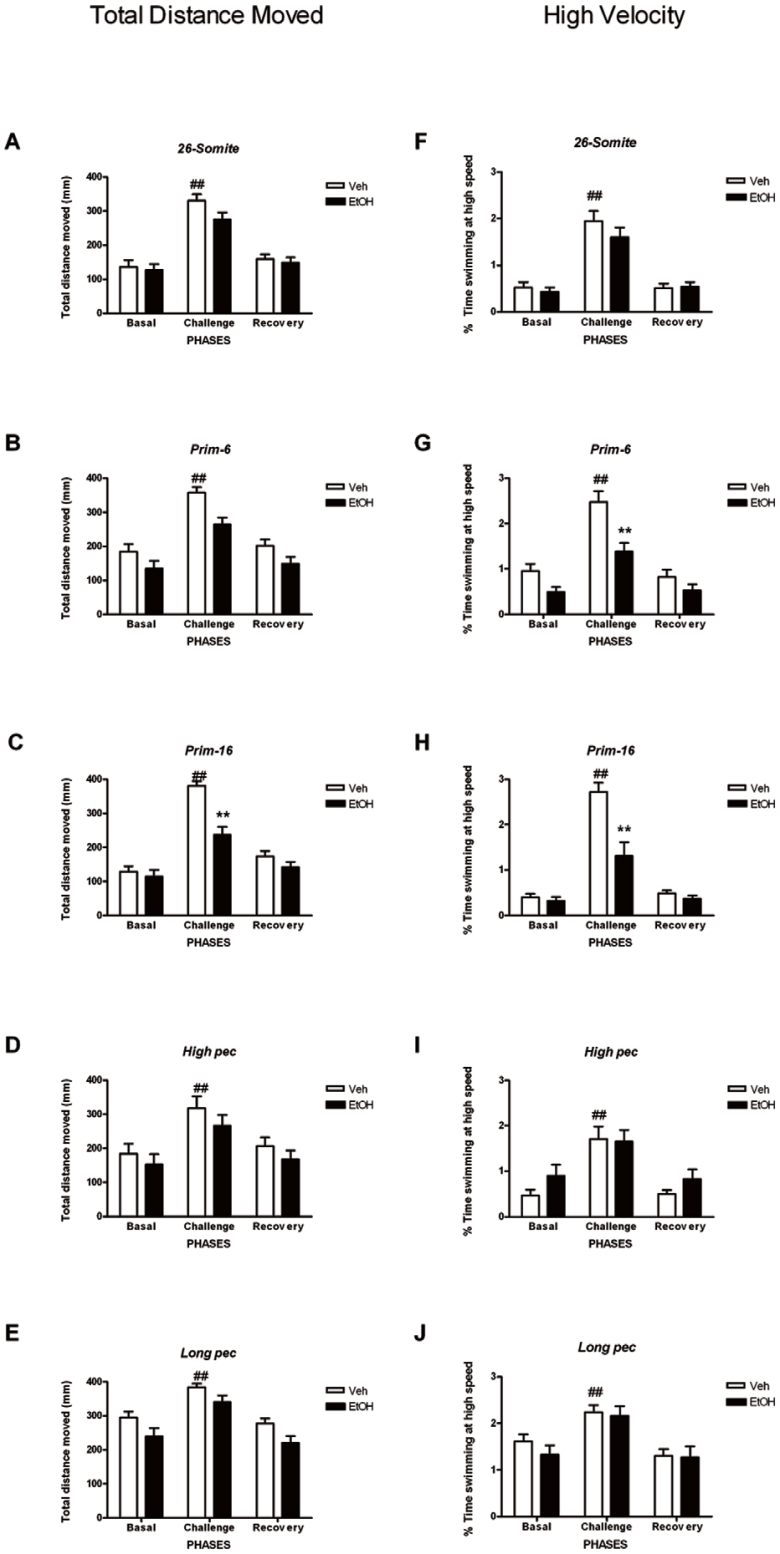
Behavioral performance in the *light-dark challenge* test. The total distance moved (**A**, **B**, **C**, **D** and **E**) and percentage of time spent swimming at high velocity (**F**, **G**, **H**, **I** and **J**) were assessed in 5 dpf larvae exposed to the *light-dark challenge* test. This shows that ethanol-treated embryos swam significantly less (reduced total distance moved) in the challenge phase (lights off) compared to the vehicle-treated controls only when ethanol exposure occurred at *prim-16* but not other stages (**C**). This finding is paralleled (**H**) by a significantly reduced ability to maintain swimming velocity at a high speed (>20 mm/sec). Furthermore, general decreases in total distance moved, regardless of the phases, are observed in ethanol-treated embryos at stages *prim-6* (**B**) and *long-pec* (**E**), suggesting general hypoactivity. This finding is also accompanied by significant reduction in the ability to maintain swimming at high velocity for larvae treated with ethanol at stage *prim-6* (**G**) but not *long-pec* (**J**). Note that stages *26-somite* (**A**) and *high-pec* (**D**) appear spared from the impact of ethanol exposure on behavioral outcome. Each error bar represents ±SEM of N = 37, 37, 32, 29, 27 embryos for vehicle and 39, 28, 26, 16, 28 for ethanol treatment at *26-somite*, *prim-6*, *prim-16*, *high pec*, and *long pec* respectively. **#** depicts differences within treatment group. *depicts differences between treatment groups. Statistical icons: ## = p<0.01, * = p<0.05, and ** = p<0.01.

Impact of ethanol exposure during specific stages of development was examined next. Total distance moved and percentage of time swimming with high velocities following exposure to the light/dark challenge test is shown in Figure 10. A two-way mixed ANOVA (Treatment [2]×Phases [3]) for repeated measures revealed a significant Treatment×Phases Interaction for stage *prim-16* [F_(1,339)_ = 10.634, *P*<0.001]. Post hoc Bonferroni test indicates that ethanol-treated embryos swam significantly less (reduced total distance moved) in the challenge phase (block 3, lights off) compared to the vehicle-treated controls only when ethanol exposure occurred at *prim-16* (*P*<0.001) but not other stages. These findings are consistent with a microphthalmia-like phenotype and altered and/or delayed locomotor development and function, which is specific to embryos treated with ethanol at *prim-16*. The latter contention is further supported by observation of a reduced ability to maintain swimming velocity at a high speed (>20 mm/sec) (F_(1.099)_ = 11.651; *P*<0.001, two-Way ANOVA, repeated measures).The post hoc Bonferroni test confirms that ethanol-treated larvae at stage *prim-16* only display a significant reduction in the percentage of time spent swimming at high speed particularly in the challenge phase (block 3, lights off) of the test (*P*<0.001; Figures 10H and M).

Furthermore, a simple main effect of treatment was observed for developmental stages *prim-6* and *long pec* [Fs_(1.0)_≥6.631, all *P*s<0.01. These findings indicate that, in general, the swimming behavior (represented here by the total distance moved) of ethanol-treated larvae was significantly dampened on all phases of the behavioral test suggesting a strong impact of ethanol on general locomotor activity. These findings were paralleled by similar observations of a reduced ability to swim at high velocities (except for *long pec*) [Fs_(1.0)_≥3.668, all *P*s<0.05]. Interestingly, larvae exposed at stages *26-somite* and *high pec* appeared to be spared from the effects of ethanol on behavioral outcome.

### Characterization of buffer

Oedema noted above in the vehicle-treated embryos was further examined in this series of experiments. To see whether the oedema in our controls was due to a problem with the buffer, or to batch variation in the embryos, we repeated the controls again and included a comparison with another buffer formulation (‘egg water’). Results are summarized in Figure S4, and show a similar pattern of low incidences of mild pericardial and yolk sac oedema with both egg water and buffer. In this additional series of 320 control embryos, no malformations of the ethanol-specific type were seen.

## Discussion

We used acute exposure (1 h pulse) because we wanted to target very specific developmental stages. The concentration of ethanol used here (10.0%) appears relatively high compared to that used in other studies (Table 1). However, it should be noticed that many of those studies involved chronic exposure. Furthermore, our HR-MAS ^1^H MRS study showed that 10% ethanol led, in intact embryos, to an internal concentration of 0.86% after 1 h, and that this value then fell to 0.0003% after 3× washing with buffer. Note that these values represent the total concentration within the space enclosed by the chorion (i.e. the perivitelline space and the embryo itself). The rather low concentrations produced by 10% exposure for 1 h do not support the view [36] that the chorion is freely permeable to ethanol.

Our acute exposure regime may be analogous in some respects to ‘binge drinking’ in humans (see [41] for a discussion of acute *versus* chronic ethanol effects in humans and animal models). Several studies have reported that binge drinking is far more damaging to the developing fetus than regular/chronic pattern of alcohol use [42]–[44].

We found that ethanol has stage-dependent effects (mortality and pharyngeal arch malformations and behavioural impariment) and stage-independent effects (microphthalmia and growth retardation) in the zebrafish. Specifically, *26-somite* stage was less sensitive to lethal effects of ethanol, while *prim-6* and *prim-16* were the most sensitive to induction of morphological malformations. We found that exposure at gastrulation stages (*50% epiboly* and *75% epiboly*) mainly resulted in high mortality. This is in contrast with studies in mice where embryos exposed at gastrulation stages were shown to develop many defects [41]. One possible explanation for this difference in response between mice and zebrafish could be our use of acute ethanol exposure, compared to the mouse studies, which used chronic exposure. Another explanation could lie in species differences in alcohol dehydrogenase, an enzyme that is not active in zebrafish gastrula approximating to *dome* and *50% epiboly* [32] but are active in mice gastrulae [45]–[47]. These enzymes metabolize ethanol to the teratogenic acetaldehyde. We are currently addressing the issue of secondary metabolites using HR-MAS ^1^H MRS.

Ethanol-treated embryos, that survived until 5 dpf, showed a wide spectrum of severity in morphological phenotypes. Our assessment of severity is to some extent subjective. Nonetheless, ee consistently found a subpopulation of survivors that were ‘resilient’, showing no malformations. Embryos that did show morphological defects, varied in severity (i.e. the number of malformations per embryo). These findings are reminiscent of the wide range of phenotypic effects, the so-called fetal alcohol spectrum [14], [15], seen in human FAS.

Our study shows that the light/dark challenge test is a useful methodology for behavioral teratogenicity in zebrafish larvae. Impairing effects of developmental exposure to ethanol on behavior were most striking in response to sudden exposure to a dark pulse when exposure occurred at stages *prim-6* and *prim-16*. The underlying cause(s) for such defects may be explained, at least in part, by developmental delays in skeletal/somatic growth. Evidence for such effects is derived from our observation of shorter body length in ethanol- relative to vehicle-treated embryos.

We also observed a general locomotor hypoactivity, regardless of changes in illumination, in ethanol- relative to vehicle-treated larvae when exposed at stages *prim-6* and *long pec*. This pattern of hypoactivity can also be due to general impairment/delay in locomotor system development and/or shorter body length incurred by ethanol treatment. In addition, it is also possible that visual impairment may contribute to the behavioral defects both in dark and light. Decreases in eye size at all stages treated (except stage *high pec*) support this contention. The fact that all larvae, regardless of treatment, responded to sudden changes in illumination argues against blindness, but it is however likely that visual efficacy/sensitivity to varying illumination might be lower in ethanol-relative to vehicle-treated larvae.

Although outside the scope of this study, long-lasting effects of developmental ethanol exposure on behavior have been reported in previous studies such as learning and memory impairment [27] and anti-social behaviors [15], [52].

It is known that ethanol exposure in fish larvae of several species (including zebrafish) can change the morphological appearance of melanocytes, at least at 7 dpf [48], [49]. Pigment cells in zebrafish also undergo aggregation or dispersion in response to environmental factors such as light, physical and chemical factors. Both neural and hormonal mechanisms are thought to regulate this process [50] and a dispersion of melanocytes has been linked to stress, that is, activation of the hypothalamic–pituitary–interregnal (HPI) axis, the teleost analogue of the hypothalamic–pituitary–adrenal (HPA) axis ([51] and refs therein). The detailed analysis of this relation is beyond the scope of this study.

Our findings of stage-specific effects can enable the search for cellular and molecular targets sensitive to ethanol and which are expressed within these stages. One cell population implicated in ethanol teratogenicity is the neural crest. These cells arise from the neural plate and migrate extensively within the embryo to give rise to elements of the craniofacial skeleton and, in mammals, elements of the cardiac septa [53], [54]. These tissues are both affected in fetal alcohol syndrome, and it is therefore reasonable to implicate damage to premigratory or migratory neural crest cells in ethanol-induced teratogenesis [55]–[57]. However, our results are not consistent with this view because at the critical period of ethanol teratogenicity, namely *prim-6* and *prim-16*, the neural crest cells of the zebrafish have already completed migration into the pharyngeal arches [58]–[66]. Thus it is possible that at least some of the hypoplasia of the pharyngeal arches seen in our study could be due to effects on postmigratory neural crest cells, in contrast to studies in chick and mouse embryos that suggest ethanol to have a major effect on migratory crest cells [9], [67]–[69]. Whether these differences are due to fundamental differences in the responses of these model species remains to be determined.

In conclusion, our use of acute, stage-specific exposure of embryos to ethanol allows stage-dependent and stage-independent effects to be identified and allows sensitive periods to be detected. This in turn allows a candidate mechanism to be more precisely defined. In the future, our large scale approach could also make it possible to identify candidate genes conferring protection against ethanol effects in the minority of individuals that show resilience.

## Supporting Information

Figure S1 **Morphometric analysis.** Illustrations showing how the morphological measurements were made in this study. **A**, 5 dpf embryo, left lateral view, showing that the body length measurement is from the tip of the lower jaw to the tip of the caudal fin. **B**, ventral view of the same embryo, showing that ‘eye size’ is the longest axial measurement of the pigmented optic cup.
(TIF)

Figure S2 **Percentage of survival at 5 dpf following ethanol exposure at various developmental stages.** A total of 384 zebrafish embryos were used as controls (vehicle) and 384 embryos were subjected to ethanol treatment at one of the eight developmental stages investigated. Survival at 5 dpf was recorded. Ethanol-induced mortality was highest when exposure occurred during *dome*, *50% epiboly*, and *75% epiboly* stages, the latter stage being the most sensitive to ethanol toxicity.
(TIF)

Figure S3 **Incidence of abnormal embryos surviving to 5 dpf after ethanol exposure at different stages.** The percentage of morphologically abnormal individuals was highest after stage *prim-6* and *prim-16* exposure. The stages *26-somite* and *long pec* were the least sensitive to ethanol-induced teratogenesis.
(TIF)

Figure S4 **Further characterization of buffers.** To investigate whether our results were influenced by some property of the buffer, 320 embryos were plated according to the standard protocols. They were raised in either ‘embryo buffer’ (used throughout this study, and based on 10% Hank's buffered saline); or another standard rearing medium, ‘egg water’ (based on ‘Instant Ocean®’; see Materials and Methods). No ethanol-specific defects, such as malformation of Meckel's cartilage or the branchial arches, were found in these experiments. This confirms that the specific malformations we saw with ethanol treatment were not due to the buffer or to a specific batch of eggs. Key: normal, no abnormalities; H, embryos with pericardial oedema only; Y, embryos with yolk sac oedema only; HY, embryos with pericardial and yolk sac oedema only.
(TIF)

Table S1 **Statistical analysis of incidence of malformations at different stages.**
(DOC)
