# Supplementary figures and images for: Large-Scale Analysis of Acute Ethanol Exposure in Zebrafish Development: A Critical Time Window and Resilience
Source: PLoS One. 2011 May 19;6(5):e20037. doi: 10.1371/journal.pone.0020037 (PMC3098763; doi:10.1371/journal.pone.0020037)

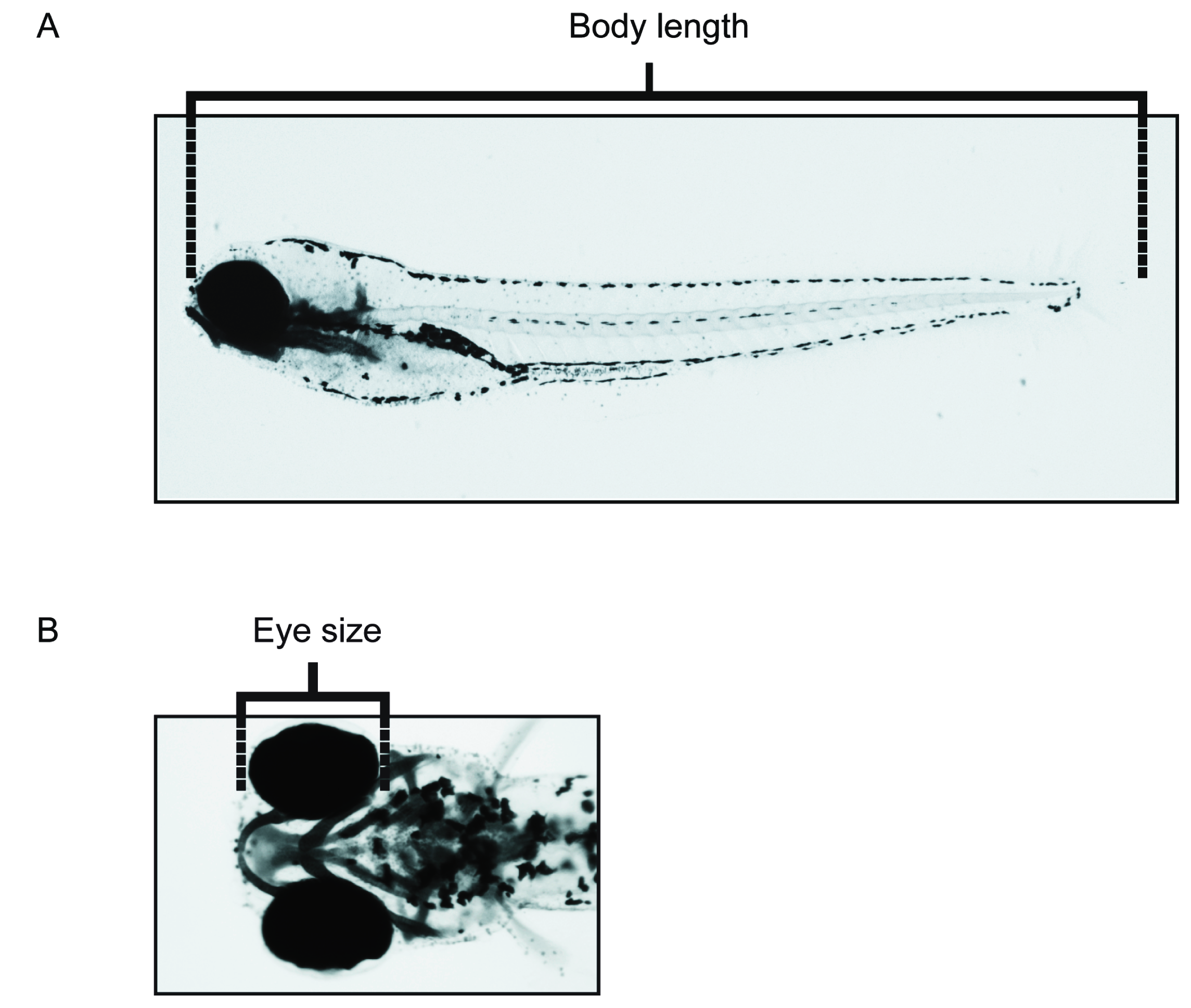

Supplement: Figure S1 — Morphometric analysis. Illustrations showing how the morphological measurements were made in this study. A, 5 dpf embryo, left lateral view, showing that the body length measurement is from the tip of the lower jaw to the tip of the caudal fin. B, ventral view of the same embryo, showing that ‘eye size’ is the longest axial measurement of the pigmented optic cup. (TIF) [file pone.0020037.s001.tif]

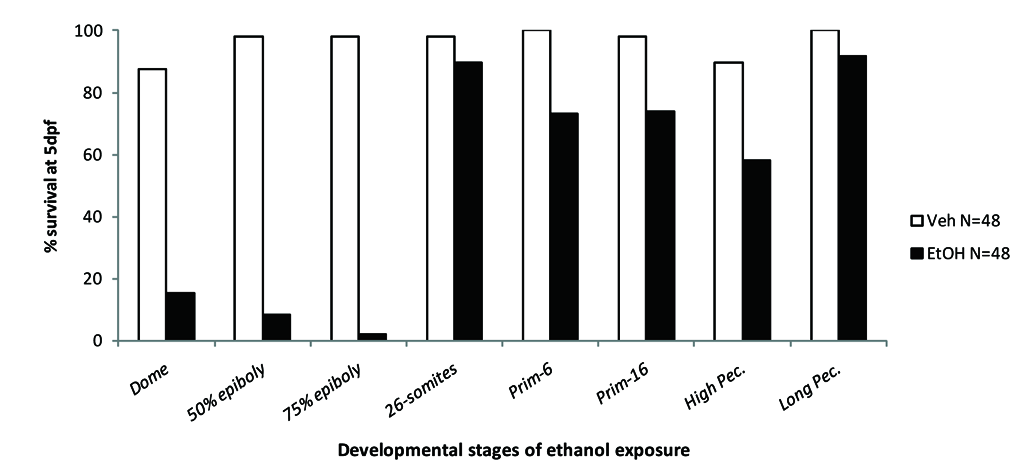

Supplement: Figure S2 — Percentage of survival at 5 dpf following ethanol exposure at various developmental stages. A total of 384 zebrafish embryos were used as controls (vehicle) and 384 embryos were subjected to ethanol treatment at one of the eight developmental stages investigated. Survival at 5 dpf was recorded. Ethanol-induced mortality was highest when exposure occurred during dome, 50% epiboly, and 75% epiboly stages, the latter stage being the most sensitive to ethanol toxicity. (TIF) [file pone.0020037.s002.tif]

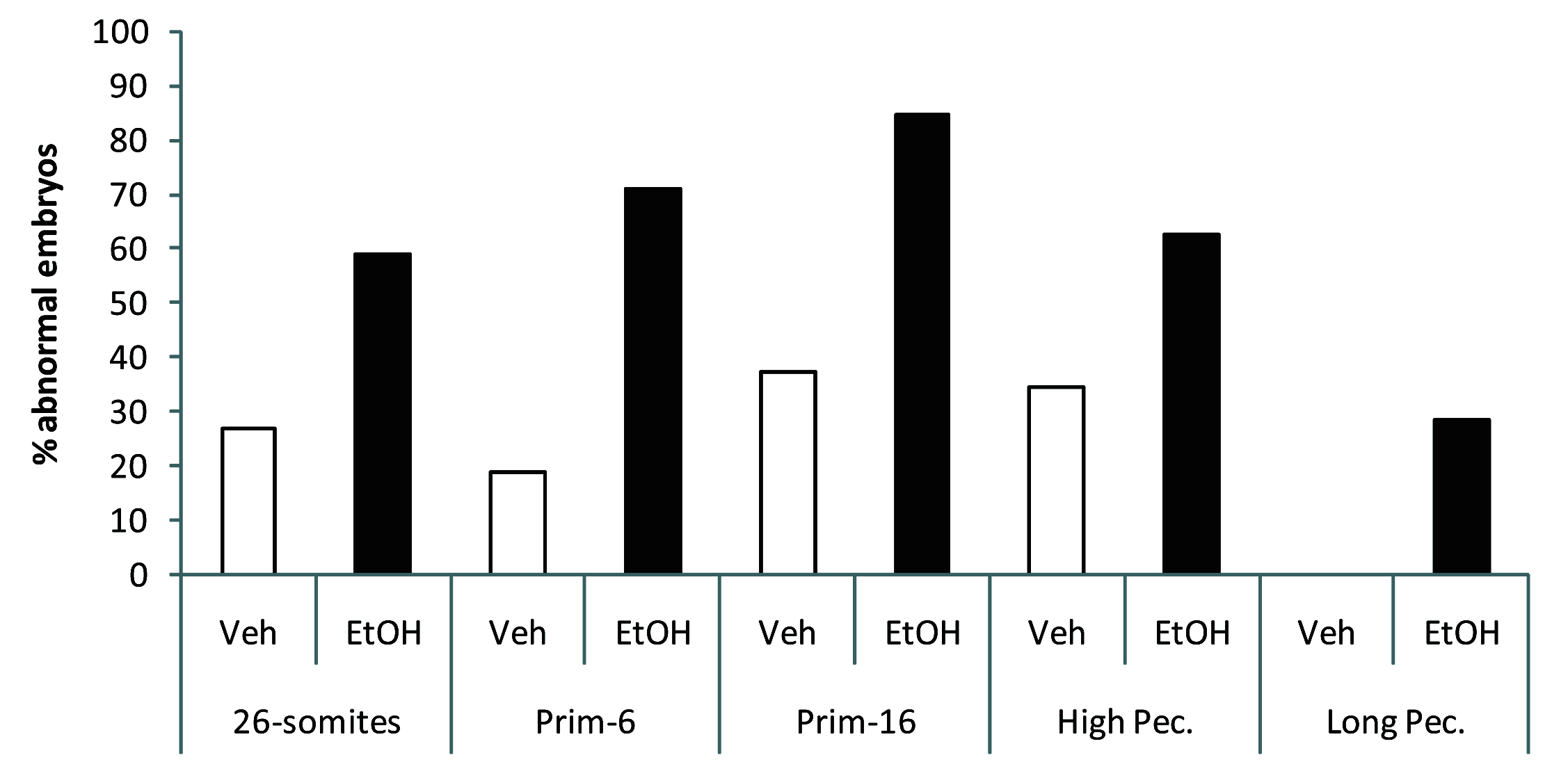

Supplement: Figure S3 — Incidence of abnormal embryos surviving to 5 dpf after ethanol exposure at different stages. The percentage of morphologically abnormal individuals was highest after stage prim-6 and prim-16 exposure. The stages 26-somite and long pec were the least sensitive to ethanol-induced teratogenesis. (TIF) [file pone.0020037.s003.tif]

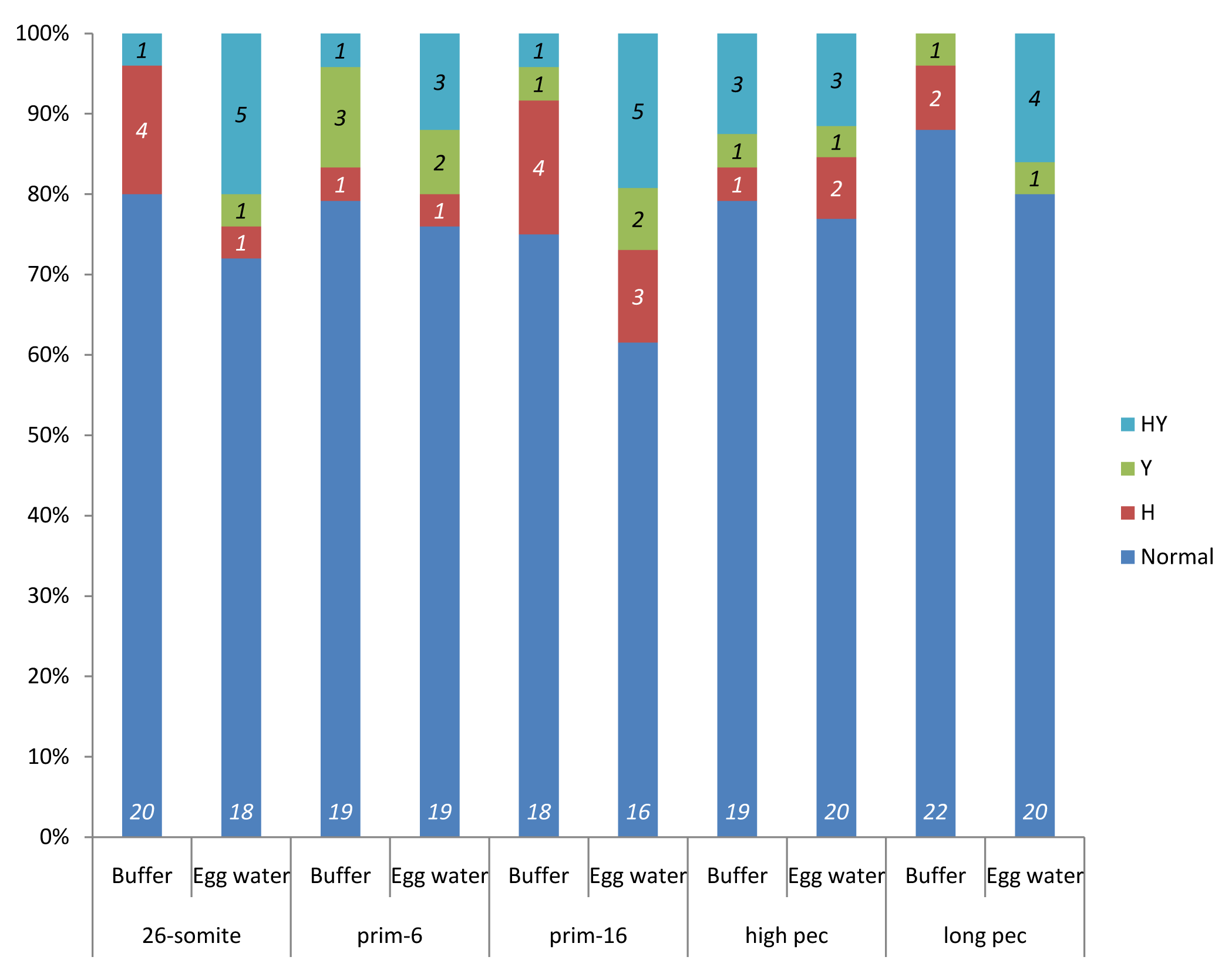

Supplement: Figure S4 — Further characterization of buffers. To investigate whether our results were influenced by some property of the buffer, 320 embryos were plated according to the standard protocols. They were raised in either ‘embryo buffer’ (used throughout this study, and based on 10% Hank's buffered saline); or another standard rearing medium, ‘egg water’ (based on ‘Instant Ocean®’; see Materials and Methods). No ethanol-specific defects, such as malformation of Meckel's cartilage or the branchial arches, were found in these experiments. This confirms that the specific malformations we saw with ethanol treatment were not due to the buffer or to a specific batch of eggs. Key: normal, no abnormalities; H, embryos with pericardial oedema only; Y, embryos with yolk sac oedema only; HY, embryos with pericardial and yolk sac oedema only. (TIF) [file pone.0020037.s004.tif]
