# Supplementary material for: Large-Scale Analysis of Acute Ethanol Exposure in Zebrafish Development: A Critical Time Window and Resilience
Source: PLoS One. 2011 May 19;6(5):e20037. doi: 10.1371/journal.pone.0020037 (PMC3098763; doi:10.1371/journal.pone.0020037)
Supplement: Table S1 — Statistical analysis of incidence of malformations at different stages. (DOC) [file pone.0020037.s005.doc]

# Supporting information

Table S1. **Statistical analysis of incidence of malformations at different stages**.

|  | **Estimate** | **Std. Error** | **z value** | **Pr(>|z|)** |
| --- | --- | --- | --- | --- |
| *high pec* (as starting level) | 1.2601 | 0.2952 | 4.27 | 0.0000 |
| *26-somite* | 0.1625 | 0.3299 | 0.49 | 0.6223 |
| *long pec* | 0.2578 | 0.3229 | 0.80 | 0.4246 |
| *prim-16* | 0.8804 | 0.2885 | 3.05 | 0.0023 |
| *prim-6* | 0.7221 | 0.2956 | 2.44 | 0.0146 |
| Eye | 0.1335 | 0.2588 | 0.52 | 0.6058 |
| Heart | 0.1643 | 0.2569 | 0.64 | 0.5225 |
| Meckel’s cartilage | -0.0741 | 0.2724 | -0.27 | 0.7855 |
| Yolk | -0.5596 | 0.3134 | -1.79 | 0.0741 |
